# Relationship between the presence of abnormal hallux interphalangeal angle and risk of ingrown hallux nail: a case control study

**DOI:** 10.1186/s12891-015-0749-1

**Published:** 2015-10-15

**Authors:** Antonio Córdoba-Fernández, Pedro Montaño-Jiménez, Manuel Coheña-Jiménez

**Affiliations:** Departamento de Podología, Universidad de Sevilla, C/ Avicena, s/n, Sevilla, Sevilla 41009, Spain

**Keywords:** Hallux valgus interphalangeus, Onychocryptosis, Interphalangeal angle, Ingrown hallux nail

## Abstract

**Background:**

Many risk factors have been identified to be associated with ingrown toenail. Internal pressure by the distal phalanx of the hallux and the second toe and external compression from the shoes has been proposed as a reason for the pathology. The main objective of the study was to analyze the existence of a correlation between the presence of pathological hallux interphalangeal angle (HIA) and risk of ingrown hallux nail.

**Methods:**

One hundred and sixty-five subjects (312 ft) were enrolled in a cross-sectional, analytical and observational case–control study. A radiographic computerized system was used to measure HIA in both groups. The angle was considered as the sum of three angles, obliquity, asymmetry and joint deviation.

**Results:**

The mean HIA in case group subjects (patients with hallux ingrown nail) was significantly higher than that obtained in control group subjects (17.39 ± 6.0° versus 13.47 ± 4.6°, *p* = .036). A total of 73.71 and 46.79 % of feet presented an angle equal or greater than 13.47° in the onychocryptosis and control group, respectively.

**Conclusions:**

The results show a correlation between the variables analysed. The presence of an HIA greater or equal than 14.5° may be a predisposing factor for developing onychocryptosis of the hallux. Clinicians treating individuals with pathology in hallux might use a baseline cutoff of HIA equal than 13.5°.

## Background

Ingrown toenail or onychocryptosis is a common problem in routine clinical practice which leads to pain and disability. Several risk factors have been identified as associated with the pathogenesis. Extrinsic risk factors including improperly trimmed nails, tight or poorly shaped shoes, abnormal nail shape, and hyperhidrosis have been associated with the pathology [1–3]. Intrinsic risk factors (congenital and heredity) have been also related to the pathology. Studies have noted anatomical abnormalities or certain forefoot shape as possible risk factors in developing ingrown toenail. Some authors have emphasized the internal pressure role that the distal phalanx on the lateral edge of the nail could play in developing onychocryptosis. Biomechanical imbalance between the great toe and the second toe in an ill-fitting shoe is the most commonly cited theory in the etiology. Some studies report a higher prevalence in people in which the first toe is shorter than the second toe (Greek foot) and also in people in whom the first and second toes are equal in length, but in whom the first metatarsal is shorter than the second (squared index minus foot type) [4, 5]. However, other authors found a higher prevalence of onychocryptosis in subjects in which the hallux is longer than the second toe (Egyptian feet types) [6].

Although many causes have been suggested based on clinical experience, current evidence suggests that the most probable cause is a combination of at least three of these factors [7, 8]. Few studies have analyzed the association between abnormal hallux interphalangeal angle (HIA) and onychocryptosis with controversial results [8, 9]. The main objective of the study was to analyze by a cross-sectional, analytical and observational case–control study the existence of a correlation between onychocryptosis and the presence of pathological hallux interphalangeal angle (HIA).

## Materials and methods

### Subjects

The required sample size was calculated using the software G*Power 3.1.3, assuming an alpha error of 5 and a beta error of 5 % to estimate the difference between 2 independent samples using one-tailed tests and with a large effect size (*d* > 0.8). The result was that a minimum of 21 cases per group was necessary. The work was carried out between February 2011 and March 2013 and was approved by the Research Ethics and Experimental Committee of the Universidad de Sevilla. The study involved a total of one hundred and sixty five subjects who were treated in the Área Clínica de Podología de la Universidad de Sevilla. The participants were patients who satisfied the inclusion criteria and gave their written informed consent to voluntarily participate in the study. The final study sample consisted of three hundred and twelve great toes from 165 subjects (63 men and 102 women; mean ± SD age, 26.3 ± 9.3 year). Both groups were homogeneous in regard to the age variable (median age was not statistically different between the groups, *p* = .08). Statistically significant differences were found regarding the distribution of sex (*p* = .008). The characteristics and distribution of the study population are listed in Table 1.

**Table 1:** General Features of patients

	Control group	Study group	Total analysed
Toes	156 (50 %)	156 (50 %)	312
Males	37	26	63
Females	44	58	102
Average age	27.19	25.35	26.27
Standard deviation	9.48	9.04	9.26
Median (range)	23 (12–50)	23 (10–50)	23 (10–50)

### Selection criteria

Eighty-four subjects were selected consecutively for the study group from among patients attending with proven unilateral or bilateral hallux ingrown toenail of all grades, and 81 for the control group selected among podiatry healthy student without history of previous onychocryptosis or another forefoot deformities. Exclusion criteria were: presence of hallux abductus valgus, obvious clinical deformity of the hind and midfoot or prior existence of osteoarticular surgery or severe trauma that could have altered the morphology of the forefoot.

### Procedure

**Fig. 1:**
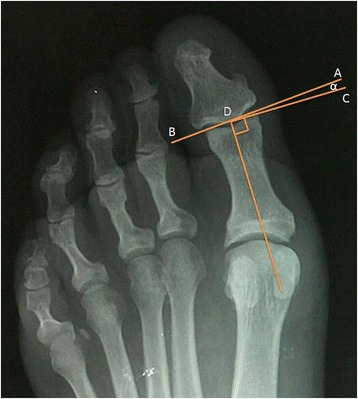
Obliquity angle: A tangent was drawn to the articular surface of the proximal phalangeal head (**a**, **b**). A perpendicular line (**c**, **d**) was then extended from the longitudinal bisection of the proximal phalanx. Obliquity is the angular relationship between this perpendicular line and the mentionated tangent

**Fig. 2:**
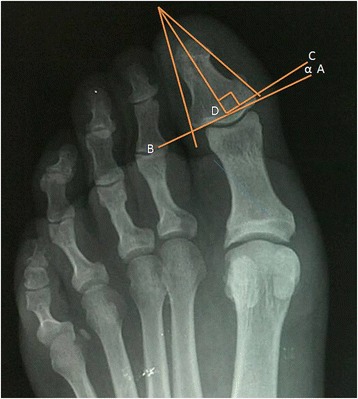
Assimmetry angle: A tangent was drawn to the articular surface of the distal phalangeal base (**a**, **b**). A perpendicular line (**c**, **d**) was then extended from the longitudinal bisection of the distal phalanx. Assymmetry is the angular relationship between this perpendicular line and the above mentionated tangent (ABC)

**Fig. 3:**
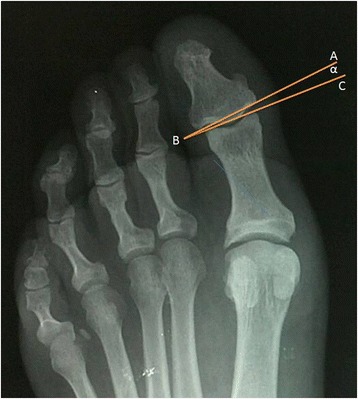
Joint deviation angle: Tangents were drawn to articular surfaces of the distal phalangeal base (**a**, **b**) and the proximal phalangeal head (**c**, **b**). Joint deviation is the angular relationship between these two tangents (ABC)

All participants were subjected to dorso-plantar x-ray (standing) view for both feet. All subjects underwent the same radiological protocol using a radiological computerized system (Kodak Point-of-Care CR 140 System®) which enabled increased image up to 300 % with greater accuracy to score the points that determine the medial axis of the metatarsal and phalangeal explored. The study protocol contained the following variables: age, sex, side, digital formula, ingrowing edge affected, first or recurrent presentation. The angles measured on the x-ray were HIA and hallux abductus valgus angle (HAVA) to rule out the existence of hallux valgus. Subjects who had HAVA equal to or greater than 15° were excluded from the study. The radiological examination was conducted by two blinded researchers to the physical findings. Measurements was performed according to the method proposed by Sorto et al. (Figs. 1, 2 and 3) such that the angle resulting angle had to be equal to the sum of the three angles obliquity, asymmetry and joint deviation. The interobserver and intraobserver reliabilities of the two researchers and the angles measurements were assessed by calculating the intraclass correlation coefficient (ICC) with confidence interval (CI) 95 %. The intraobserver reliability was measured with an interval of one week. Any value above 13.4° for HIA was considered abnormal. Data were analyzed using SPSS Version 21 (SPSS Inc, Chicago, IL).

## Results

The results of the measurements determined that the average value of the HIA from the study group was significantly higher than the control group (*p* = .036) (Table 2). The percentage of subjects with HIA higher than 13.47° was 73.71 and 46.79 % for the study and control group, respectively. The percentage of individuals in the study group with HIA ≥ 15° was 69.72 %. No statistically significant differences were recorded in regard to the average HIA in the study group according to sex (Table 3). The maximum average value of HIA in the study group was recorded for the interval of subjects aged between 21–30 years old and the minimum average value aged 41–50 years old (Table 4) with statistically significant differences between age intervals (*P* < .05). The Anova test found statistically significant differences in regard to the HIA according to the age range in both groups (*p* < .001). The Scheffe multiple comparisons test determined the existence of statistically significant differences in regard to the HIA (*p* = .002) recorded between the two age ranges 21–30 and 41–50 (Table 5). The ICC for intraobserver reliability was 0.96 and for interobserver reliability was 0.92. In regard to the frequency of nail edge affected, 50.64 % of those from the study group had both folds affected, 42.95 just the lateral edge and just 6.41 % for the medial edge. Aχ^2^-Pearson correlation coefficient with continuity correction revealed there is no relationship between the existence of a pathological HIA (≥13.47°) and the affected nail edge (Table 6). In regard to the kind of digital formula recorded for those from the study group, the Egyptian foot digital formula was the most prevalent with 64.10 % of individuals followed by 25 % of individuals with a Greek foot formula and 10.90 % with square foot formula.

**Table 2:** Comparison of the mean HIA between groups (*N* = 312 toes, 165 patients)

Degrees	Control group	Onychocryptosis	P
Median (minimum, maximum)	(12.73, 14.20)	(16.44, 18.33)	
Mean ± SD	13.47 ± 4.66	17.39 ± 6.00	.036^a^
Typical mean error	.374	.481	
95 % CI (lower limit, upper limit)	(12.73-14.20)	(16.44-18.33)	

^a^Student *t* test; CI (95 %)

**Table 3:** Distribution of the mean of HIA in the study group according to sex

	Mean ± SD	Typical mean error	Lower-upper limit
Males (63 subjects)	15.59 ± 6.31	.42	14.49-16.16
Female (102 subjects)	15.33 ± 5.30	.50	14.59-16.58

*N* 156 toes, 84 patients, *P* .280; CI (95 %)

**Table 4:** Means distribution of HIA values for both groups included according to age group analysed

	Study group	Control group
Age intervals		
10-20 years	15.64 ± 4.57	13.95 ± 3.58
21-30 years	18.67 ± 6.72	14.71 ± 5.19
31-40 years	18.17 ± 4.21	10.92 ± 4.06
41-50 years	14.69 ± 6.82	11.74 ± 4.16

Data are presented as mean ± SD (95 % confidence interval)

**Table 5:** Comparisons of the mean HIA performed within the different age groups in the study group

Variable	Mean of difference	*P* value^*^
Age group of 21-30		
VS 10-20	2.02	.69
VS 31-40	2.36	.100
VS 41-50	4.02	.002

^*^ Scheffe test of multiple comparisons; CI (95 %)

**Table 6:** Contingency table between abnormal HIA and nail edge affected

			Lateral	Nail edge medial	Total
HIA	<13.47º	Recount	4	37	41
		Expected frequency	2.6	38.4	41
	≥13.47º	Recount	6	109	115
		Expected frequency	7.4	107.6	115
Total		Recount	10	146	156
		Expected frequency	10	146	156

χ^2^-Pearson’s Correlation Coefficient: *P* = .517

## Discussion

Traditionally hallux abductus interphalangeus or valgus deformity has been defined as an increase in the HIA of greater than 10° [10, 11]. Existing prior studies have revealed that the existence of an abnormal HIA may be related to the risk of suffering from ingrown hallux nail. In a case–control study on a sample of 80 patients, Darwish et al. analyzed the HIA and HAVA in subjects with onychocryptosis in the hallux (cases) and compared this with 80 healthy subjects (controls). As in our work, the percentage of abnormal HIA in the study group was also significantly higher (85 %) compared to just 25 % in the control group (*p* < .005). In the same way, the authors also found statistically significant differences (*p* < .005) when they analyzed the HIA of patients with prior episodes of ingrown hallux nail in just one of the toes and compared this with the healthy contralateral toe that had never suffered from onychocryptosis. The study concluded that the existence of abnormal HAVA and HIA represents a risk factor for the pathogenesis of onychocryptosis of the hallux [9].

Conversely, in a retrospective study performed by Kose et al. the x-rays of 81 patients with onychocryptosis were studied and compared to those of 100 healthy subjects. The HAVA, HIA and intermetatarsal angles (IMA) were measured; no statistically significant differences were found between the groups in regard to any of the three angles assessed. The average HIA value obtained in the case group was slightly higher but without any statistically significant differences (11.9° ± 4.1 vs. 12.0° ± 5.1; *p* = .123). The authors conclude that the study results do not reveal a correlation between forefoot misalignments and the existence of onychocryptosis, and they suggest that management of the pathology should not be based on correction of forefoot misalignments but rather managing the pathology itself [8].

In both studies, the authors set out as normal HIA values those that do not exceed 10° and that has been considered a reference value for adults subjects in most studies performed [10, 12, 13]. However, this value does not correspond to observational studies performed on the general population; in fact, other authors establish mean reference values above 10°. Barnett in a study performed on 346 x-ray from feet in 173 British healthy subjects (mean age: 21.4 year) with shoes set out as mean HIA values for men 13° ± 3.72 for the left foot; 13.1° ± 2.34 for the right foot; and in women 13.3° ± 3.54 for the left foot and 13.5° ± 3.53 for the right foot [14]. Sorto et al. determined a mean HIA value in x-rays from 100 ft in subjects of different ages and for both sexes of 13.4° and they considered feet with HIA above this value as pathological [15].

The main aim of our work was to determine whether there is a direct correlation between the existence of ingrown hallux nail and the presence of a pathological HIA. Therefore, we have used a more precise system of measuring HIA than that used in prior studies. The mean HIA value obtained in the study group was significantly higher than the control group. The results indicate to us that it is difficult for a subject with HIA values ≥ 15.5° not to suffer or be a future sufferer of onychocryptosis in the great toe. This highlights the impact of abnormal HIA on the onset of ingrown hallux nail. The authors recommend appropriate follow-up of these subjects to assess need for realignment surgery.

In the study performed by Darwish et al., the measurement technique used may have caused significant margins of error in measuring the angles studied, because these were performed with manual markers and rulers [9]. In the same way, in the study by Kose et al. the angles were measured by a collaborator outside the study, but without specifying the method used for this measurement [8]. The results obtained in this study are supported by the measurement technique used to measure the different angles. Barnett attributed the angular relationship between the longitudinal bisections of the distal and proximal phalanges of the great toe to two factors: an oblique orientation of the articular surface of the head of the proximal phalanx (obliquity), and an asymmetrical form of the distal phalanx itself (asymmetry) [14]. For Sorto et al. and Duke et al. the HIA value is in turn the sum of the three angles; angle of obliquity, asymmetry and combined joint deviation (angular relationship between the articular surfaces of the base of the distal phalanx and head of the proximal phalanx) which have been appropriately taken into consideration to perform this work [15, 16]. Farber et al. revealed that measuring certain angles on x-rays using a digital system is fully valid and leads to improving inter and intraobserver reliability in comparison with the use of the analogous technique by means of goniometer and pencil [17]. The program used in this work has the property of increasing the image size by zoom without distorting it; this enables reducing the margin of error when we mark the points that serve as a reference to determine the mean axis of the phalanges.

The possible relationship of HIA with age was part of a secondary aim that we had not set in this study. The age range with a higher HIA value was 21–30 and the lower value 41–50. In regard to sex no statistically significant differences were found in regard to HIA values recorded in both groups. In regard to the digital formula, we found that 64 % of the patients in the study group had a Greek foot digital formula. These results coincide with those reported by Viladot and are substantially different to those obtained by Günal et al. [4, 18]. We agree with the first author who found more pathological changes in the forefoot of patients with Egyptian feet. For the interphalangeal hallux deformity not only does this lead to deformity in the transverse plane but also in the frontal plane, which also causes more compromise of space with footwear in people with Egyptian foot digital formula. We consider therefore that individuals with Greek foot or square digital foot are less at risk of suffering from forefoot pathologies because of the footwear as a consequence of the lower vulnerability of these to the standard triangular form of the shoe tip which facilitates better accommodation of the foot to the footwear.

In regard to the frequency of nail edge affected, in the study of Darwish et al. the 85 % of patients in the study group had affected the lateral edge [9]. Kose et al. excluded patients in their study group with medial or bilateral ingrowing nail edge [8]. Günal et al. did not report any information regarding affected percentage of edges although reports that patients treated with a spacer between the first and second toes healed in about three weeks [4]. We have found that more than half of the patients in our study group had affected both nail edges. Overall the lateral edge was affected by more than 93 % of cases.

The findings of this work are based on by a cross-sectional, analytical and observational case–control study, however, the limited population and the presence of others common risk factor related with the pathology may have introduced a selection bias. Future research should focus on validating the results of this study, a prospective study looking at the feet of an unaffected populations and then observing whether patients with increased HIA developed ingrown toenails would be definitive.

## Conclusion

The results of our study have revealed the correlation existing between the main variables; it is verified that patients that present increased HIA values are more likely to develop ingrown hallux nail. We can therefore state that among the possible etiological causes of onychocryptosis, the existence of a pathological HIA plays a substantial role in the genesis of the pathology. The presence of an HIA greater or equal than 14.5° may be a predisposing factor for developing ingrown hallux nail. The results indicate to us that it is difficult for a subject with HIA values ≥ 15.5° not to suffer or be a future sufferer of onychocryptosis. This approach can enable clinicians to better design treatment programs to minimize the risk. Clinicians treating individuals with ingrown hallux nail may use this baseline cutoff of HIA as one of the indicators of risk. The results of this study suggest that in subjects with HIA angle ≥ 15.5° affected by other associated risk factors should be followed. If the pathology recurs after conservative or surgical procedures in the nail unit, it will highlight the need for alignment procedure.

## Abbreviations

HIA: Hallux interphalangeal angle
HAVA: Hallux abductus valgus angle
ICC: Intraclass correlation coefficient
CI: Confidence interval
IMA: Intermetatarsal angle
